# In Vitro Evaluation of Biological Activities of Canes and Pomace Extracts from Several Varieties of *Vitis vinifera* L. for Inclusion in Freeze-Drying Mouthwashes

**DOI:** 10.3390/antiox11020218

**Published:** 2022-01-24

**Authors:** Anca Pop, Catalina Bogdan, Ionel Fizesan, Sonia Iurian, Rahela Carpa, Cecilia Bacali, Laurian Vlase, Daniela Benedec, Mirela L. Moldovan

**Affiliations:** 1 Department of Toxicology, Faculty of Pharmacy, “Iuliu Hațieganu” University of Medicine and Pharmacy, 6 L. Pasteur Street, 400349 Cluj-Napoca, Romania; anca.pop@umfcluj.ro; 2 Department of Dermopharmacy and Cosmetics, Faculty of Pharmacy, “Iuliu Hațieganu” University of Medicine and Pharmacy, 12 I. Creanga Street, 400010 Cluj-Napoca, Romania; catalina.bogdan@umfcluj.ro (C.B.); mmoldovan@umfcluj.ro (M.L.M.); 3 Department of Pharmaceutical Technology and Biopharmacy, Faculty of Pharmacy, “Iuliu Hațieganu” University of Medicine and Pharmacy, 41 V. Babes Street, 400012 Cluj-Napoca, Romania; sonia.iurian@umfcluj.ro (S.I.); laurian.vlase@umfcluj.ro (L.V.); 4 Department of Molecular Biology and Biotechnology, Faculty of Biology and Geology, “Babes-Bolyai” University, 1 M. Kogalniceanu Street, 400084 Cluj-Napoca, Romania; rahela.carpa@ubbcluj.ro; 5 Department of Prosthodontics and Dental Materials, “Iuliu Hațieganu” University of Medicine and Pharmacy, Clinicilor Street 32, 400006 Cluj-Napoca, Romania; cecilia.bacali@yahoo.com; 6 Department of Pharmacognosy, Faculty of Pharmacy, “Iuliu Hațieganu” University of Medicine and Pharmacy, 12 I. Creanga Street, 400010 Cluj-Napoca, Romania; dbenedec@umfcluj.ro

**Keywords:** pomace, canes, extract, cytocompatibility, antioxidant, anti-inflammatory, antimicrobial, freeze-drying, mouthwash, *Vitis vinifera*

## Abstract

In this study, the biological activities of four extracts from *Vitis vinifera* by-products: two pomace extracts, white (WPE) and red (RPE), a canes extract (CE), and their combination (CoE), were evaluated, to be included in freeze-drying mouthwashes formulations. The cytocompatibility and anticancerous potential of the four extracts were tested on three cancerous cell lines, as well as the cytoprotective activity against nicotine-induced cytotoxicity and the antioxidant potential determined on a human gingival fibroblasts (HGF) cell line. Additionally, the anti-inflammatory activity and the antimicrobial activity against several microorganisms from the oral microbiome were tested. Freeze-dried mouthwashes with CoE were prepared and characterized, both as lyophilizates and after reconstitution. The four tested extracts showed the highest cytotoxicity on MDA-kb2 cell line. The antioxidant potential was demonstrated for WPE, RPE, CE, and CoE, both in non-stimulated and H_2_O_2_ stimulated conditions. The four extracts reduced the levels of proinflammatory cytokines (IL-6, IL-8, and IL-1β) in a dose-dependent manner, confirming their anti-inflammatory activity. The antimicrobial activity of tested extracts was shown against pathogenic bacteria from the oral microbiome. Mouthwashes of CoE with poloxamer-407, xylitol, and different ratios of mannitol were prepared by freeze-drying leading to porous formulations with interesting mechanical properties and reconstitution times.

## 1. Introduction

*Vitis vinifera* L. (grapevine) is a plant belonging to the Vitaceae family, which is extensively used in the food and wine industry. This plant and its by-products, mainly grape skins and seeds, pomace, leaves, and canes, are important sources of polyphenols with potential biologic activity such as cardioprotective, antioxidant, anticancer, anti-aging, anti-inflammatory, and antimicrobial effects [1]. Among these polyphenols, the flavonoids are the most abundant, mainly in grape seeds but also in the grape skin, both materials being part of the grape pomace. It has been estimated that over 70% of the total polyphenols remain in grape pomace. Furthermore, ellagic acid is deemed to be an important active compound in nutraceuticals due to the antimutagenic, anticarcinogenic, antioxidant, and hepatoprotective properties [2].

The antioxidant activity of polyphenols extracted from *Vitis* spp. is often reported in the scientific literature, being attributed mainly to their metal chelating properties, to their ability to scavenge the free radicals, to reduce the hydroperoxide formation or to inhibit the lipid oxidation [3]. The antioxidant activity of *Vitis* polyphenolic extracts is ascribed rather to the total active ingredients content than to a single component [4]. Due to their ability to reduce the oxidated LDL in plasma, to protect the mitochondrial system against oxidative stress induced by hydrogen peroxide, to decrease the oxidative stress in serum, and to protect against DNA damage the grape polyphenols are being administered orally. Other applications of polyphenolic extracts from *Vitis vinifera* L. include their external use for medicinal purposes in skin cancer prevention [5] or for cosmetic purposes in oral care, to reduce the oxidative stress which is involved in periodontitis pathogenesis [6], in skin care for antiaging purposes [7], in skin photoprotection [8], or to reduce skin pigmentation [9].

The effectiveness of polyphenols from grape extracts in reducing chronic inflammation is also mentioned in several scientific publications, as a result of their ability to modulate the inflammatory pathways along with the decrease of the reactive oxygen species levels [2]. Thus, the suppression of several proinflammatory cytokines, particularly the transcription factors NF-kB, IL-6, IL-8, and IL-1β, by various *Vitis* extracts was reported [2,10].

An important purpose of oral care products is to control the dental plaque, a biofilm in which a complex microbial community develops. Among the antimicrobial substances used to control dental plaque, chlorhexidine is currently used, along with triclosan, essential oils, or stannous and zinc salts. Undesirable effects like tooth staining, diarrhea, vomiting, but also dysbiosis, and bacterial resistance to antiseptic agents, namely chlorhexidine and triclosan, were observed [11,12]. To reduce the risk of selecting multidrug-resistant strains, it is important to find alternative sources of antimicrobial agents, such as phytochemical compounds, which are proved to be effective alternatives to synthetic substances [12]. In this regard, a rich source of natural active ingredients is found in the winery industry where a high amount of residues containing bioactive ingredients content results [13].

Up to date, the use of grape products in oral care has been extensively reported in the scientific literature. The polyphenols from grape products and by-products can suppress the growth of microorganisms in dental plaque, one of the most important causes of dental caries and periodontal disease. *Streptococcus mutans* is considered the main microorganism identified in the early development of dental caries, being identified in about 90% of isolates from human caries [14]. Another plaque-mediated disease, the periodontal disease is associated with the presence of anaerobic microorganisms *Porphyromonas gingivalis*, *Tanerella forsythia*, and *Treponema denticola*, which form the “red complex”. Other pathogens involved in the progression of the disease are *Prevotella* spp., *Fusobacterium* spp., and *Parvimonas micra* which form the “orange complex”. *Porphyromonas gingivalis* is considered the main etiological factor which disrupts the relationship between normal and pathogen microbiota of oral cavity, initiates the destructive cascade and leads to inflammation and bone destruction, as it was shown in a murine model for periodontal disease. *Porphyromonas* *gingivalis* specific CD4^+^ T cells predict the onset of the disease, but also its progression, determine the clonal expansion kinetics and the cytokine expression. *Treponema* spp. *Fusobacteriun* spp. are pathobionts rather than trigger pathogens [15]. The antimicrobial efficacy of grape pomace extract against several pathogens such as *Staphylococcus aureus*, *Escherichia coli*, *Candida albicans*, and *Pseudomonas aeruginosa* was previously reported [16]. In addition, the antimicrobial efficacy of leaves and tendrils extracts from *Vitis vinifera* L. against *Staphylococcus aureus*, *Escherichia coli*, *Candida albicans*, and several oral pathogens such as *Streptococcus mutans*, *Enterococcus faecalis*, *Porphyromonas gingivalis*, and *Klebsiella* spp. was demonstrated [10].

The present study aimed to evaluate the bioactivities of *V. vinifera* by-products, by investigating the anticancerous, antioxidant, anti-inflammatory, and antimicrobial activity of white pomace extract (WPE), red pomace extract (RPE), and canes extract (CE) from *V. vinifera*. The second step of the study focused on the preparation of an oral care product, namely a lyophilized mouthwash containing *V. vinifera* extracts that may be used in the prevention and treatment of different oral health conditions. Thus, the mechanical structure and the reconstitution time of the lyophilizates were investigated. The viscosity and texture profile of reconstituted mouthwashes were further investigated and compared with a commercially available mouthwash. Following the analysis of the results, an optimal formulation was chosen.

## 2. Materials and Methods

### 2.1. Plant Material

The pomace and canes of some varieties of *Vitis vinifera* were obtained from the experimental fields of Murfatlar winery company (44°10′25″ N 28°24′30″ E, Constanta County, Romania). The red pomace (RP) sample was a mixture of equal parts of vegetable product from Pinot Noir, *Feteasca neagra*, Cabernet Sauvignon, and *Mamaia varieties*. The white pomace (WP) sample was a mixture of equal parts of Muscat Ottonel and Sauvignon Blanc varieties, while the canes (C) sample was a mixture of equal parts of all the above varieties. The plant material was dried at room temperature, while being aerated by palletizing to facilitate water removal and avoid microorganism contamination, once every 24 h. Further, it was ground (RC-21 Electroarges, Arges, Romania) for 5 min and passed through a 200 µm sieve (Retsch, Haan, Germany) [17]. 

### 2.2. Chemicals

Dimethyl sulfoxide (DMSO) (≥99%), hydrogen peroxide (H_2_O_2_) 30% solution, N-acetyl-l-cysteine (≥99%), phosphate buffer, resazurin, 2,7 dichloro-fluorescein diacetate (DCFH-DA), and Lipopolysaccharides isolated from *E. coli* were purchased from Sigma Aldrich (Schnelldorf, Germany). Dulbecco’s modified Eagle medium (DMEM) was purchased from Gibco (Paisley, UK), and fetal bovine serum (FBS) from Sigma Aldrich (Steinheim, Germany). ELISA kits for the quantification of IL-6 and IL-8 were acquired from Biolegend (San Diego, CA, USA), while IL-1β kit was obtained from Biogems (Westlake Village, CA, USA). To test the antimicrobial activity, the following growth media purchased from Oxoid, UK, and prepared according to the manufacturer’s instructions were used: Nutrient agar and Müeller–Hinton agar.

The materials used in the second phase of lyophilized mouthwash preparation were mannitol Parteck^®^ M 200 (Merck, Darmstadt, Germany), Poloxamer 407 (PX) (Sigma-Aldrich, St. Louis, MO, USA), xylitol (Elemental, Oradea, Romania), and distilled water.

### 2.3. Extract Preparation

The extraction conditions were previously optimized through the Design of Experiments methodology [17]. Briefly, the dry and ground (particle size < 200 µm) plant material (RP, WP, and C) was used for reflux extraction at 80 °C in an aqueous ethanol solution of 50% (*V*/*V*), at a solid: solvent ratio of 1:10 (*w*/*V*). After cooling, the samples were filtered through filter paper in a graduated flask, then centrifuged (1930× *g*) for 20 min and the supernatants were recovered [17]. So finally, three extracts were obtained, coded RPE, WPE, and CE, and a fourth combined extract (CoE) was prepared as a mixture of equal parts of the previous three and subsequently used to investigate the antimicrobial activity.

For the preparation of the lyophilized mouthwash, the combined extract (CoE) was concentrated through rotary evaporation (Hei-VAP Advantage Rotary evaporator HL/G1 (Heidolph, Germany) under reduced pressure at 50 °C and 80 rpm until 33.0313% of the initial weight, and further used for the formulation of the mouthwashes. 

The extracts used for cell culture experiments were concentrated following the above-mentioned steps and further lyophilized. For each lyophilized extract, a 100 mg/mL stock solution was prepared in dimethyl sulfoxide (DMSO) that was further diluted to obtain the working solutions. These solutions were diluted in cell culture medium to obtain the desired concentrations, ranging from 400 to 10 µg/mL. 

### 2.4. Characterization of the Extracts

The characterization phenolic of the WPE, RPE, and CE extracts using LC/MS method was previously reported [17]. The polyphenolic compounds were determined with LC/MS/MS in negative ion mode, and identified by comparison of their retention times and the MS spectra with those of the standards, using the same chromatographic parameters (e.g., reversed-phase HPLC columns, mobile phase with methanol: 0.1% acetic acid and a binary solvent gradient, flow rate = 1 mL/min, injection volume = 5 µL, column temperature = 48 °C with combined detection: UV (330 nm, 370 nm) as in our previous publication. The experiments were performed on an Agilent 1100 HPLC Series system equipped with: degasser, binary gradient pump, autosampler, column thermostat, and UV detector that was coupled with an Agilent Ion Trap 1100 SL mass spectrometer (LC/MSD Ion Trap VL). DataAnalysis and ChemStation software (Agilent Technologies, CA, USA) were used for data collection and processing. The retention times were determined using reference standards and were based on the mass spectrum for each compound. Spiking samples with a solution containing each polyphenol (10 µg/mL) was used for accuracy check. For identification of compounds, their retention times and the recorded ESI-MS spectra were compared with those of standards, which were obtained under identical working conditions. The method of external standard was employed for the quantification of polyphenols in each extract and the calibration curves for a five-point plot were linear in the range 0.5–50 µg/mL (R2 > 0.999) [17].

### 2.5. Cell Culture

The cancerous cell lines A549 (human lung adenocarcinoma), T47D-KBluc (human breast cancer), and MDA-kb2 (human breast cancer) were purchased from American Type Culture Collection (ATCC, Manassas, VA, USA). A549 cells were maintained in DMEM medium supplemented with 10% FBS, T47D-KBluc cells in RPMI-1640 supplemented with 10% FBS and MDA-kb2 cells in Leibovitz’s L-15 Medium supplemented with 10% FBS. Normal human gingival fibroblasts (HGF) were purchased from CLS Cell Lines Service, Eppelheim, Germany, and were maintained in DMEM supplemented with 10% FBS. Except for the MDA-kb2 cells that were cultured without additional CO_2_, cells were routinely cultured at 37 °C in a humidified incubator with 5% CO_2_ supplementation and the medium was changed every 2–3 days. Cells were routinely harvested at 70–80% confluence for subculturing or further use in experiments.

### 2.6. Cytocompatibility and Anticancerous Potential

The cytotoxicity of the four extracts was evaluated by Alamar Blue (AB) assay using the protocol previously described [18] after an exposure of 24 h to the extracts. Cells were seeded in 96 well plates at an adjusted cell density (10.000 A549 cells/well, 20.000 T47D-KBluc cells/well, 20.000 MDA-kb2 cells/well and 2500 HGF cells/well) to reach a confluency of 70–80% at the end of the experiment, and left to attach to the substrate for 24 h. Cells were further exposed to increasing concentrations (10–400 μg/mL) of the WPE, RPE, CE, and CoE for another 24 h. After the exposure, cells were washed two times with PBS and further incubated with a 200 µM solution of resazurin for 2–4 h. The conversion of resazurin (non-fluorescent) to resorufin (fluorescent) by metabolically active cells was measured at λ_excitation_ = 530/25; λ_emission_ = 590/35, using Synergy 2 Multi-Mode Microplate Reader. The experiments included three biological replicates, each one including six technical replicates and negative control (cells exposed to culture medium containing 0.2% DMSO). The results were expressed as relative values compared to the negative control (100%). 

### 2.7. Antioxidant Potential

The ability of the WPE, RPE, CE, and CoE to reduce the oxidative stress in HGF-1 cells was evaluated using the ROS sensitive dye 2′,7′-Dichlorofluorescin Diacetate (DCFH-DA) as previously described [19]. Briefly, HGF cells were exposed for 24 h to non-toxic concentrations of WPE, RPE, CE, and CoE (100, 200, and 300 µg/mL) and further loaded with 50 µM DCFH-DA in Hanks’ Balanced Salt Solution (HBSS) for 2 h. To quantify ROS in stimulated and non-stimulated conditions, the cells were either exposed to 250 µM H_2_O_2_ in HBSS or HBSS for 2 h. The conversion of CFH-DA to the fluorescent compound dichlorofluorescein (DCF) by ROS was optically measured using Synergy 2 Multi-Mode Microplate Reader at an λ_excitation_ = 485/20 and λ_emission_ = 528/20. The potency of the WPE, RPE, CE, and CoE to mitigate the induction of oxidative stress was compared to a standard antioxidant (20 mM solution of N-Acetyl Cysteine (NAC) treatment).

### 2.8. Cytoprotective Effects against Nicotine-Induced Cytotoxicity

The cytoprotective effects of the WPE, RPE, CE, and CoE against nicotine-induced toxicity were measured using the AB assay described above based on a previously reported protocol [10]. HGF cells were co-exposed for 24 h and 48 h to a mixture of extracts at 100, 200, and 300 µg/mL and nicotine at the calculated IC_50_ values (8 mM and 5 mM at 24 h and 48 h, respectively). After the incubation period, cells were washed with PBS and further incubated with a resazurin solution of 200 µM for 4 h. For each condition, three biological replicates, each one with six technical replicates were performed and included a negative control (medium containing 0.2% DMSO). The results are presented as relative values compared to the negative control (100%)

### 2.9. Anti-Inflammatory Potential

The WPE, RPE, CE, and CoE anti-inflammatory activities were firstly evaluated by quantifying three pro-inflammatory cytokines (IL-8, IL-6, and IL-1β) from cell culture supernatants using ELISA assays. HGF cells were simultaneously exposed to 100 ng/mL LPS and three non-cytotoxic concentrations of the WPE, RPE, CE, and CoE (100, 200, and 300 µg/mL) for 24 h. To rule out an additive cytotoxic effect of LPS in the presence of the WPE, RPE, CE, and CoE extracts, the cytotoxic effects of the mixtures on HGF cells were first evaluated. Following the 24 h exposure to the mixtures of LPS with extracts, the concentrations of IL-8, IL-6, and IL-1β were measured using commercially available ELISA Kits from a standard curve fitted based on a four-parameter logistic curve according to the manufacturer’s instructions.

### 2.10. Antimicrobial Activity

The following microorganisms were chosen to carry out the test: *Streptococcus mutans* ATCC 25175, *Porphyromonas gingivalis* ATCC 33277, *Enterococcus faecalis* ATCC 29212, *Escherichia coli* ATCC 25922, *Staphylococcus aureus* ATCC 25923, *Klebsiella* sp., and *Citrobacter* sp., which can be components of the normal oral microbiota or the periodontopathic microbiota. The antimicrobial activity was tested through a qualitative diffusimetric method, adapted to the disk/well method, which is considered a reliable method with large applicability in clinical practice to test the efficacy of most antimicrobial substances [20]. According to this method, substances that diffuse in a culture medium create a circular area where the concentrations of antibacterial substances are higher in the center and lower to the periphery. All experiments were performed in a vertical laminar flow air hood Steril Helios (Bionova, Italy). Each bacterial strain was grown for 24 h on Nutrient Agar medium. From each microbial strain, a dilution of 0.5 McFarland in sterile saline water was made, which was spread over the entire surface of the Muller Hinton Agar medium. After 30 min of drying at 37 °C, sterile swabs were applied in the wells and then 80 µL of each of the test samples were added, consisting of the extracts obtained from *Vitis vinifera* L. pomace and canes and a control sample—the solvent used for extraction. Incubation was performed at 37 °C for 18–24 h, thereafter the diameter of the inhibition zone was measured. The diameter of the inhibition area is correlated with the sensitivity of bacterium to the tested extracts [21]. 

### 2.11. Preparation of the Freeze-Dried Mouthwashes

Mouthwashes were designed as freeze-dried structures obtained by the lyophilization of CoE solutions that were meant to be reconstituted with a certain volume of water before use. Five solutions (coded F1 to F5) were prepared by using CoE, PX, mannitol, and xylitol. The CoE solutions were poured into 2 mL and 4 mL PVC blister sockets. The amount of CoE needed for each blister socket (0.10 g) was calculated from the concentration that showed the highest anti-inflammatory and antioxidant effects during the in vitro studies. PX, a nonionic surfactant was added at two different CoE:PX ratios, 1:1 and 2:1 respectively, as shown in Table 1. Mannitol was added at two different ratios, CoE:mannitol 1:1 and 1:2, respectively. One sample (F5) included xylitol at a ratio of 4:1 xylitol:CoE. The mouthwashes were prepared by physical mixing of the PX and CoE followed by the addition of an aqueous solution containing mannitol. The mixtures were gently stirred until the complete dispersion and homogenization. Precise volumes (2 mL or 4 mL, according to Table 1) of each sample were poured into the blister alveolae and freeze-dried using a lab-scale VirTis Advantage Plus freeze-drier (SP Scientific, Gardiner, NY, USA) under the following regime: the samples were subjected to a fast-freezing step and then kept at −55 °C for 12 h. The primary drying was performed at −25 °C for 24 h and vacuum of 0.2 mbar, followed by secondary drying at 20 °C for 12 h. Afterward, the obtained lyophilizates were extracted from alveolae and analyzed.

### 2.12. Characterization of the Freeze-Dried Mouthwashes

The textural characteristics of freeze-dried mouthwashes were determined using Brookfield CT3 Texture Analyzer (Brookfield Engineering, Middleboro, MA, USA). The samples were carefully removed from alveolae and placed on the rigid test surface (TA-BT-KIT fixture). The measurements were performed during a compression test by using the TA10 probe. During the test, the cylinder probe performed the compression of the sample with a constant speed of 0.1 mm/s and a load of 10 g, until a penetration depth of 5 mm. For each formulation, the determinations were performed in triplicate and the mean value ± standard deviation was reported. The texture profile data was collected using TexturePro CT Software V 1.9 (Brookfield Engineering, Middleboro, MA, USA) and the following parameters were calculated: hardness of the sample measured as the load value recorded at highest peak during compression, the rigidity of the sample measured at 3 mm depth, and fracturability calculated as the load value recorded at the first fracture. Generally, these characteristics are indicative of the brittleness of the samples.

The reconstitution time was measured by placing the sample in 10 mL distilled water, the necessary volume for the reconstitution of the mouthwash. The time elapsed until the complete disintegration of samples when no solid residue was perceived, was measured using a digital stopwatch. The analyses were performed in triplicate and the average times ± standard deviation was reported. 

### 2.13. Characterization of the Reconstituted Mouthwashes

Brookfield CT3 Texture Analyzer (Brookfield Engineering, Middleboro, MA, USA) equipped with the TA-DEC probe and TA-BT-KIT was used to determine the reconstituted mouthwash texture. The purpose of the instrumental texture analysis was to provide objective measurements that indicate sensory attributes of the formulation [22], the variations in texture characteristics being the result of the variations in the composition of the samples [23]. The texture measurements of the reconstituted mouthwashes consisted of hardness, adhesive force, and stringiness length determinations. A commercially available alcohol-free formulation (Sensodyne Mouthwash Cool Mint^®^—F6) containing the same nonionic surfactant was also analyzed to establish the desired quality profile of the final product. Back extrusion test was performed by applying a load of 1 g with a constant speed of 1 mm/s while the probe penetrated the sample to a target distance of 20 mm. During the compression of the sample, several parameters that define the peak force and area of work were recorded. The negative part of the force–time graph describes the sample adhesiveness. The measurements were performed in triplicate and the mean value ± standard deviation was reported.

The viscosity of the reconstituted mouthwashes was determined at 23 ± 0.2 °C and 200 rpm by using a rotational rheometer Brookfield DV-III Ultra (Brookfield Engineering, Middleboro, MA, USA) equipped with spindle LV-1. The measurements were performed in triplicate and the mean value ± standard deviation of each determination was reported.

### 2.14. Statistical Analysis

The data are presented as mean values ± standard deviation (SD) of at least three biological replicates. The normally distributed data sets were analyzed using one-way analysis of variance (ANOVA) with a post hoc Holm–Sidak test. Unless stated otherwise, data analyses and graphical representation were done using the SigmaPlot 11.0 computer software (Systat, Software Inc., Chicago, IL, USA). Results with a *p* values less than 0.05 were considered statistically significant.

## 3. Results and Discussion

### 3.1. Overview on Phenolic Compounds Identified in Vitis vinifera Pomace and Canes

Table 2 summarizes the polyphenolic composition of the WPE, RPE, and CE extracts. Phenolic acids such as gallic, protocatechuic, and syringic acids were found in all samples together with catechin and epicatechin [17].

### 3.2. Cytocompatibility and Anticancerous Potential

**Figure 1:**
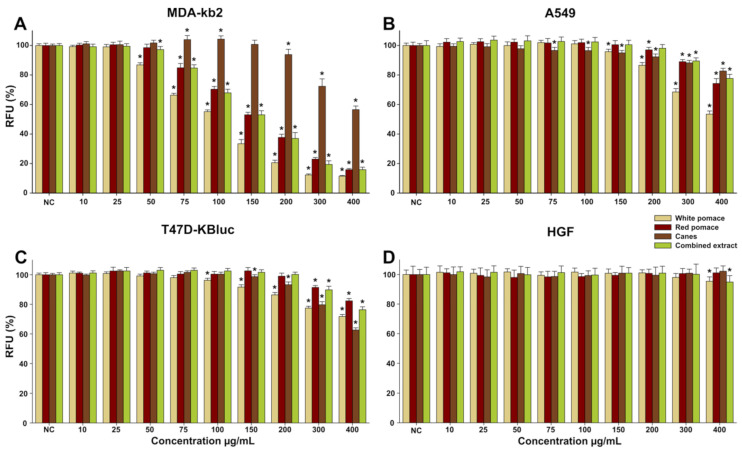
Cytotoxicity of the WPE, RPE, CE, and CoE was evaluated using Alamar Blue assay on the cancerous cell lines MDA-kb2 (**A**), A549 (**B**), and T47D-KBluc (**C**) and normal human gingival fibroblasts (**D**) after a 24 h exposure. The results are presented as the means ± standard deviations of three biological replicates, each one including 6 technical replicates. The values were expressed as relative values compared to the negative control (DMSO 0.2%) (100%). Asterisks (*) indicate statistically significant differences in comparison with negative control (ANOVA + Holm–Sidak post hoc test at *p* < 0.05).

The anticancerous potential of the *Vitis vinifera* extracts was evaluated on three cancerous cell lines including mammary (MDA-kb2 and T47D-KBluc) and pulmonary (A549) derived cancerous cell types in parallel with normal human fibroblasts. In comparison with the normal cell type where only WPE and CoE significantly decreased the viability at the highest tested dose of 400 µg/mL, higher toxicity was observed in cancerous cells (Figure 1). Similar to these results, we previously reported that hydroalcoholic extracts from leaves and tendrils from the same species are not cytotoxic towards normal cells at concentrations of up to 400 µg/mL [10]. These differences in terms of toxicity of the *Vitis vinifera* extracts between normal and cancerous cell phenotypes were also reported in other papers, the higher toxicity towards the cancerous phenotype being stipulated to be the result of an interference in mitochondrial membrane potential that further leads to apoptosis in cancerous cells [24,25]. Moreover, De Sales et al. reported that a short exposure of cancerous cells (HepG2) to an anthocyanin-rich grape pomace extract leads to increased mitochondrial respiration and decreased glycolytic metabolism, irrespective of observed cytotoxicity, while a long term exposure is associated with cellular death by necrosis in cancerous cells, but not in normal cells [26]. 

The highest cytotoxicity of the extracts was observed in the case of MDA-kb2 cell line, where the WPE, RPE, CE, and CoE significantly decreased the viability starting from a dose of 50 µg/mL, respectively. The observed decrease in the cellular viability was dose-dependent, the highest dose tested decreasing the cellular viability below 20% in the case of WPE, RPE, and CoE and below 60% for CE. Interestingly, at intermediate doses, CE induced a hormetic response in MDA-kb2 cell line, the viability at the doses of 75 and 100 µg/mL being approximately 105% of that measured for the negative control (Figure 1A). The higher sensitivity of the MDA-kb2 cell line to the *Vitis vinifera* extracts was recently reported by Liozzo et al. that evaluated the anti-proliferative activity of *Vitis vinifera* leaves extracts and obtained an IC_50_ value of approximately 30 µg/mL for the most promising extract [27]. In the case of A549 and T47D-KBluc cell lines, the anticancerous activities of the extracts were modest, WPE displaying the highest activity from the extracts evaluated (Figure 1B,C).

### 3.3. Antioxidant Potential

**Figure 2:**
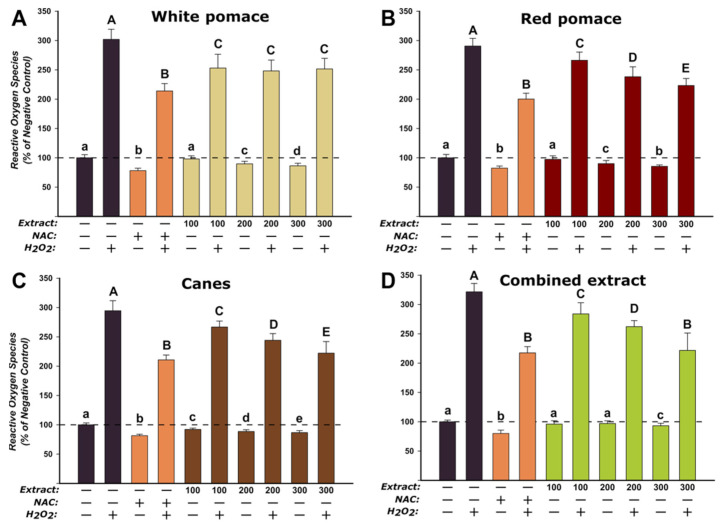
Antioxidant effects of WPE (**A**), RPE (**B**), CE (**C**), and CoE (**D**) were evaluated using DCFH-DA assay on HGF. Cells were treated with the extracts (100, 200, and 300 µg/mL) or NAC (20 mM) for 24 h and further exposed to 50 µM DCFH-DA. The antioxidant potential was measured after a 2 h incubation in the presence and absence of 250 µM H_2_O_2_ (stimulated/non-stimulated conditions). The data are expressed as relative means ± standard deviation of three biological replicates, each one including 6 technical replicates. The values were expressed as relative values compared to the negative control (DMSO 0.2%) (100%). Different letters (a–e refers to comparisons in non-stimulated conditions, while A–E refers to comparisons in stimulated conditions) indicate statistically significant differences (ANOVA + Holm–Sidak post hoc test at *p* < 0.05).

The antioxidant effects of the *V. vinifera* extracts were evaluated on the normal fibroblast cells at three concentrations (100, 200, 300 µg/mL) that did not induce statistically significant toxicities after an exposure of 24 h. The assay was performed in two conditions, namely H_2_O_2_-stimulated and non-stimulated conditions. In non-stimulated conditions, similar to what was observed for the standard antioxidant NAC, exposure to the extracts resulted in a statistically decrease in ROS that was dose-dependent (Figure 2). In stimulated conditions, exposure of HGF to H_2_O_2_ alone led to an approximately threefold increase in the concentration of ROS, while pre-incubation with the antioxidant NAC partially mitigated this increase. Similar to the non-stimulated conditions, the WPE, RPE, CE, and CoE presented an antioxidant potential, exposure to the extracts resulting in a decrease in ROS, that except for WPE was dose-dependent. These results endorse previous data published by our group where we reported the antioxidant properties of these extracts in abiotic systems, more specifically DPPH and FRAP assays [28]. Recently, Milinčić et al. reported that an extract from grape pomaces from the Prokupac red grape variety possess antioxidant activities in cellular cultures, the data indicating that this effect is positively correlated with the content in anthocyanins and flavonoids, mainly quercetin, kaempferol, syringetin, malvidin, and petunidine [29]. Antioxidant properties of extracts derived from grape pomace were also reported by Maluf et al., exposure to these extracts offering cellular protection against the oxidation promoted by peroxide exposure in normal fibroblasts [30]. Cytoprotection and antioxidative properties of grape skin extracts were also reported by Fernández-Fernández, exposure to the pre-digested extract of intestinal and immune cells resulting in protection against the formation of ROS and nitric oxide [31]. Using DCFH-DA assay, grape skin extract has been shown to possess antioxidant potential in isolated mitochondria [32]. Overall these findings are congruent with the data in the scientific literature, various extracts from different parts of *V. vinifera* displaying antioxidant properties in cellular and non-cellular assays [33,34,35,36], thus indicating the potential cosmetic use in preventing/mitigating skin aging associated with oxidative stress in dermal cells.

### 3.4. Cytoprotective Agent against Nicotine-Induced Cytotoxicity

**Figure 3:**
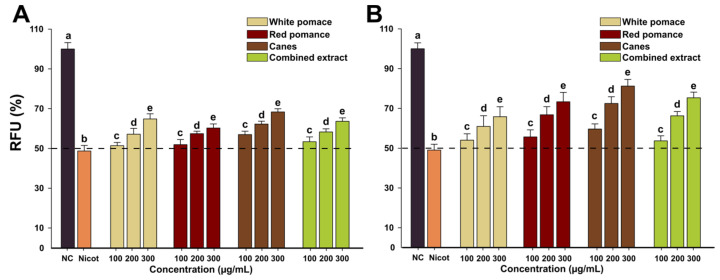
Cytoprotective effects of WPE, RPE, CE, and CoE against nicotine-induced cytotoxicity were analyzed after 24 h (**A**) and 48 h (**B**) exposure to 100, 200, and 300 µg/mL of the extracts in combination with nicotine (8 mM and 5 mM at 24 h and 48 h, respectively) using AB assay. The values are expressed as mean ± standard deviation (SD) of three biological replicates. Different letters (a–e) represent the significant differences in mean cellular viability (ANOVA + Holm–Sidak post hoc test at *p* < 0.05 level of significance).

Nicotine represents one of the major factors involved in the initiation and progression of inflammatory diseases [37], current studies indicating that it has a pernicious effect by potentiating the pro-inflammatory effects of LPS, the main factor involved in periodontitis [38,39,40]. In this regard, the cytoprotective effects of the *V. vinifera* extracts against nicotine cytotoxicity in human gingival fibroblasts were evaluated. The cytoprotective effect of three non-toxic concentrations of the extracts (100, 200, 300 µg/mL) was evaluated at 24 h and 48 h using AB assay. For all the extracts tested, a cytoprotective dose-dependent effect was observed starting from the lowest tested concentration of 100 µg/mL at both time points evaluated (Figure 3). CE displayed the highest potency in mitigating the nicotine-induced toxicity, at the highest tested dose decreasing the nicotine toxicity by approximately 40% and 60% at 24 h and 48 h, respectively. In accordance with the current results, we recently reported the cytoprotective effects of two extracts from leaves and tendrils of *V. vinifera* after an exposure of 24 h [10]. Compared with the previously published results where the highest concentration tested decreased the nicotine-induced cytotoxicity by approximately 20% for both extracts, WPE, RPE, CE, and the CoE had a more significant effect. Moreover, for all the extracts an improved protective effect was observed after an exposure of 48 h, indicating the potential usage of these extracts in reducing the deleterious effects of oral nicotine exposure during chronic exposure (Figure 3B). The observed effects are related to the rich content of the extracts in polyphenols, flavonoids, and caffeic acid derivatives, compounds with antioxidant properties that can mitigate the increase in ROS induced by nicotine exposure [41,42,43,44,45]. In this regard, Desjardins et al. reported that epigallocatechin-3-gallate displayed cytoprotective effects at low doses in oral epithelial and fibroblasts cells exposed to cytotoxic concentrations of nicotine [46].

### 3.5. Anti-Inflammatory Potential

**Figure 4:**
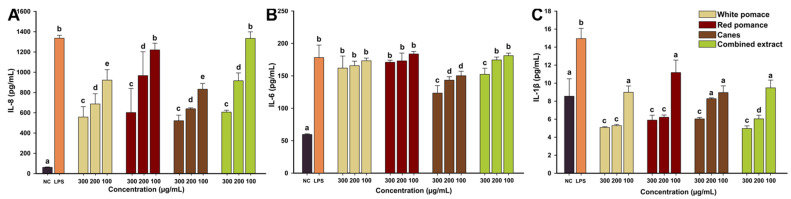
The anti-inflammatory potential of WPE, RPE, CE, and CoE. The quantity of IL-8 (**A**), IL-6 (**B**), and IL-1β (**C**) was measured from cellular supernatants after a 24 h exposure to the extracts (100, 200, and 300 µg/mL) in combination with 100 ng/mL LPS. Data are presented as mean ± standard deviation of four biological replicates. Different letters (a–e) indicate statistically significant differences in quantities of cytokine measured (ANOVA + Holm–Sidak post hoc test at *p* < 0.05 level of significance).

The anti-inflammatory effects of the *V. vinifera* extracts were evaluated by measuring the levels of three pro-inflammatory cytokines (IL-8, IL-6, and IL-1β) in an inflammation-induced cellular model. The pro-inflammatory response was induced in HGF by exposing the cells to a non-cytotoxic dose of lipopolysaccharides (LPS) isolated from *E. coli*. Exposure of HGF to 100 ng/mL LPS induced a robust inflammatory response, the levels of all three pro-inflammatory cytokines displaying a statistical increase (Figure 4). From the three cytokines evaluated, the level of IL-8 had the most striking increase, with the concentration increasing by more than 22-fold in comparison with the negative control. After the successful implementation of the LPS-induced inflammation, the anti-inflammatory potential of WPE, RPE, CE, and CoE, at non-cytotoxic doses, was evaluated. 

All four extracts displayed anti-inflammatory properties, which in most of the cases were dose-dependent. Starting from the lowest dose of 100 µg/mL, WPE and CE significantly reduced the level of IL-8, while for RPE and CoE a significant decrease in IL-8 was observed only from the intermediate dose of 200 µg/mL (Figure 4A). At the highest tested dose of 300 µg/mL, all extracts displayed similar anti-inflammatory effects, decreasing the level of IL-8 by approximately 60%. In the case of IL-6, only exposure to CE and the highest dose CoE resulted in a statistically decrease in the levels of this cytokine. Even though a decreasing trend in the level of IL-6 was observed for WPE and RPE no statistical difference was present (Figure 4B). In the case of IL-1β, all extracts had an anti-inflammatory potential starting from the lowest dose evaluated. At this dose, the levels of IL-1β were not different from the levels of the negative control. Interestingly, at the intermediate and the highest doses, the extracts significantly decreased the IL-1β concentration below the level of the negative control (Figure 4C). Based on the levels of these pro-inflammatory cytokines, CE displayed the highest anti-inflammatory potential, while CoE, obtained from the mixture of white and red pomace and canes, had an intermediary anti-inflammatory effect. In previous research, our group reported that the hydroalcoholic extracts of leaves and tendrils from *V. vinifera* possess anti-inflammatory properties, decreasing the levels of pro-inflammatory cytokines elicited by the LPS exposure in human gingival fibroblasts [10].

The results obtained are in accordance with data from the scientific literature, different extracts from *V. vinifera* displaying in vitro and in vivo anti-inflammatory effects [47,48,49,50].

### 3.6. Antimicrobial Potential 

Oral care cosmetics such as mouthwashes are intended to cleanse the oral cavity and to control halitosis by providing an agreeable taste and odor. Those products may contain ingredients with antimicrobial activity in order to control the microbial load in the oral cavity, and therefore they can be effective in the prevention of several oral conditions such as the supragingival plaque, gingivitis, the incipient stage of periodontal diseases, or caries [51]. Due to the built-up resistance to the antibiotics used in dentistry, the plant extracts gained major importance in preventing bacterial oral growth and colonization. It is also well known that different plants and natural compounds drive a substantial antimicrobial activity against different microorganisms [52,53]. In this sense, the polyphenolic compounds may be used for counteracting the growth of antibiotic-resistant pathogenic bacteria [54]. The antimicrobial activity of grape pomace was extensively investigated and it is ascribed both to flavonoid content (flavanols and flavonols) and also to the nonflavonoid content (phenolic acids and stilbenes) [55].

The diameter of the inhibition area for the tested extracts varied according to the tested microbial strain, while the control sample showed no inhibition area (Table 3). 

It was observed that CE exhibited the most important inhibition of *Streptococcus mutans* ATCC 25175 and *Enterococcus faecalis* ATCC 29212 strains, WPE and RPE showed a good inhibition of *Porphyromonas gingivalis* ATCC 33277 strain, RPE also showed a good inhibition for the following bacterial strains *taphylococcus aureus* ATCC 25923, *Escherichia coli* ATCC 25922 and *Klebsiella* spp., while WPE had the greatest inhibition diameter for *Citrobacter* spp. A good inhibition of all tested strains was observed for the CoE, suggesting that a broad-spectrum antimicrobial activity can be obtained by combining the individual extracts.

### 3.7. Characterization of the Freeze-Dried Mouthwashes through Texture Analysis

Out of the different extracts and ratios that were tested for the anti-inflammatory, cytoprotective and antioxidant effects, the highest overall benefits were shown for CoE at a concentration of 300 µg/mL. Therefore, CoE was carried on to the second part of the study and included into the freeze-dried mouthwash formulations. The CoE content was calculated so that the concentration of the reconstituted formulations in the designated volume of water, of 10 mL, would be 300 µg of dried extract per Ml.

Freeze-dried products are usually obtained by the lyophilization of a solution, or a suspension containing an active ingredient and display high porosity which goes along with fast reconstitution, but also with significant friability that sometimes impairs manipulation. So, mannitol was added as a filler and also for its cryoprotective effects at ratios of 2.5% (F1, F3, F4, F5) and 5% (F2) [56]. It was expected to have an impact on the mechanical resistance, on reconstitution time, and to give a pleasant taste. PX was considered for its solubilizing properties and suspension stabilization properties, at ratios of 1.25% (F3) and 2.5% (F1, F2, F4, F5). Ten percent of the xylitol was added to the F5 solution due to its cariostatic properties and to its potential to reinforce the enamel. The same content was ensured for formulations F1 and F4, but the alveolae volume decrease from 4 mL to 2 mL which was expected to determine changes in the mechanical profile and reconstitution time.

**Figure 5:**
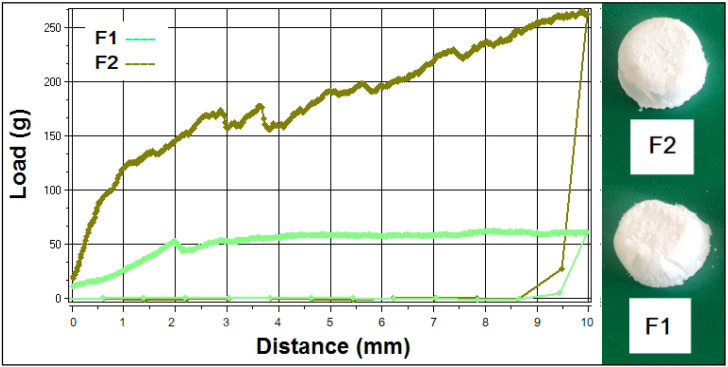
The mechanical profile of formulations F1 and F2 expressed as load vs. distance (**left**), associated to their physical appearance (**right**).

Freeze-drying led to five porous formulations of 25 mm diameter × 15 mm height (F1, F2, F3, and F5) and of 18 mm diameter × 14 mm height (F4) which were characterized through texture analysis, for hardness, rigidity, and fracturability (Table 4). Different compositions yielded structures with various mechanical properties. The increase in mannitol ratio in formulation F2 compared to F1, led to a firm and stiff structure, that was easy to extract from the blister sockets, and to a significant increase in hardness. As shown in Figure 5, the curve corresponding to F2 reaches a higher value of the load and displays more fractures, but at much higher loads when compared to F1. F2 also revealed the highest value of fracturability that measures the resistance to fractures, an important parameter during the packaging and transport of the product. 

F3 contained lower ratios of PX when compared to F1 and had very porous and brittle structures that made the extraction from alveolae difficult. Consequently, a significant decrease in hardness and fracturability was recorded, so apparently, PX also had a positive effect on the mechanical properties. F4 had a similar composition to F3, but a volume of 2 mL was poured in each alveolae. This led to slightly increased hardness, rigidity, and fracturability, yet no significant differences when compared to F1. So, the volume of the freeze-dried structures does not seem to have an important impact on the mechanical characteristics. F5 displayed the highest values that define the mechanical profile of the samples; adding 10% of xylitol led to significantly higher mechanical parameters, but the products presented tacky surfaces, thus difficult to handle.

### 3.8. Characterization of the Reconstituted Mouthwashes

Reconstitution time was tested in the volume of water necessary for one application, of 10 mL, and varied between 4.82 and 30.56 s. Significant differences were found between all formulations F2–F5 when compared to F1, because except for F1, all formulations disintegrated rapidly, in less than 10 s. That indicates that the changes performed on the initial formulation, like mannitol increase or adding xylitol led to faster reconstitution, but also consolidated structures with increased mechanical strength. 

The composition of the lyophilizates determines the texture parameters which are good predictors of the sensory characteristics of the product. So, the texture analysis was performed on the reconstituted samples and finally compared to the ones of a commercial mouthwash. As shown in Table 5 there were no significant differences between the texture parameters of formulation F1 when compared to formulations F2–F5. Samples’ hardness, measured as the load at the highest peak during the compression cycle, varied between 13.70 and 17.00 g. The adhesive force, recorded as the negative peak when the probe is detached from the sample, varied between 7.70 g and 10.00 g. Adhesive properties could be important for the remanence of the product on the oral mucosa so higher adhesive forces for the F1–F4 formulations were considered favorable. Stringiness was measured as the travel distance of the probe during the negative force area and varied between 0.53 and 0.54 mm [22,23].

The low influence of the formulation factors was expected because the added excipients were all highly soluble in water with no impact on texture, but a slight increase of viscosity was obtained when adding higher ratios of polyols [57]. Moreover, formulations F2 and F5 had similar characteristics when compared to the commercial formulation, both in terms of texture and viscosity, but considering the difficulties in the manipulation of the freeze-dried formulation F5, F2 was chosen as an optimal product. 

Attempting to formulate solid mouthwashes is not a novel approach, several patents and scientific articles reported the preparation of soluble, dispersible, effervescent tablets used as oral rinses after a prior water dissolution [58,59]. Some patents were filed for solid mouthwashes containing herbal extracts, out of which *Vitis vinifera* extracts were selected for their vasoconstrictor and tonic effects and were compressed with surfactants, disintegrants and/or effervescent agents [60,61]. The authors claimed that the active ingredients presented better stability as compared to the solutions, the products were less voluminous and had no alcoholic content. Most of these products used dry extracts, thus the preparation processes comprised several stages: extraction, evaporation/concentration/drying of extracts, followed by granulation or compression, out of which the drying process could be critical for the composition of the final product due to the thermal sensitivity of some bioactive compounds. Out of the drying methods, freeze-drying is known to ensure high biocompound preservation into the final product, due to the low operating temperatures and high vacuum [62]. This effect was demonstrated on the high recovery of polyphenols when the herbal products were freeze-dried, but also when dry extracts or encapsulated bioactives were obtained by lyophilization [63]. Lately, freeze-drying was also used as a preparation method for pharmaceutical, nutraceutical, and cosmetic products whose porous structures ensure high dissolution rates and fast reconstitution [64].

Up to this point of the study, the effect of the extracts was demonstrated in vitro, and an optimal formulation was chosen for its potential application, a freeze-dried mouthwash. Of course, this optimization strategy has several limitations, conclusions cannot be established outside the studied formulation domain; however, the best results were obtained for formulation F2, so further studies will follow to evaluate the in vivo effects of the product. 

## 4. Conclusions

*Vitis vinifera* by-products represent a valuable source of polyphenols that may lead to various health benefits. The current study evaluated the bioactivities of several *V. vinifera* by-products, namely white pomace, red pomace and canes extract. The anticancerous potential of the four extracts was evaluated on three cancerous cell lines including mammary and pulmonary-derived cancerous cell types, in parallel with normal human fibroblasts. The highest cytotoxicity of the tested extracts was observed in the case of MDA-kb2 cell line, while in the case of A549 and T47D-KBluc cell lines, the anticancerous activities of the extracts were modest. All the extracts displayed an antioxidant potential, anti-inflammatory properties, and an improved protective effect against nicotine-induced cytotoxicity. In addition, the tested extracts had an observable antimicrobial effect on several strains existing in normal oral microbiota or periodontopathic microbiota. The results obtained during in vitro study supported the inclusion of the combined extract, prepared as a mixture of equal parts of the individual extracts, in a lyophilized mouthwash formulation. Following the characterization of the five formulations, an optimal freeze-dried mouthwash with good structural and mechanical properties was chosen. Up-to-date, a limited number of lyophilized compositions for oral care have been reported. As an element of novelty, the current research provides scientific evidence to argue the suitability of these innovative mouthwash formulations in the prevention and the treatment of different oral health conditions, such as periodontal disease. 

## Figures and Tables

**Table 1:** Composition of the solutions used for the freeze-dried mouthwashes.

Code	Alveolae Volume	Composition Expressed as g/Alveolae (*w*/*V*, %)				
		CoE	PX	Mannitol	Xylitol	Distilled Water
F1	4 mL	0.10 (2.50)	0.10 (2.50)	0.10 (2.50)	-	added to the alveolae volume (up to 100%)
F2	4 mL	0.10 (2.50)	0.10 (2.50)	0.20 (5.00)	-	
F3	4 mL	0.10 (2.50)	0.05 (1.25)	0.10 (2.50)	-	
F4	2 mL	0.05 (2.50)	0.05 (2.50)	0.05 (2.50)	-	
F5	4 mL	0.10 (2.50)	0.10 (2.50)	0.10 (2.50)	0.40 (10.00)	

**Table 2:** Polyphenolic composition of WPE, RPE, and CE [17].

Polyphenolic Compounds	WP (µg/g)	RP (µg/g)	C (µg/g)
Gallic acid	128.19 ± 0.93	146.87 ± 1.13	39.57 ± 0.30
Protocatechuic acid	57.17 ± 0.24	34.88 ± 0.12	34.88 ± 0.12
Caftaric acid	<0.02	<0.02	<0.02
Gentisic acid	<0.02	<0.02	-
Catechin	539.14 ± 1.86	561.93 ± 4.07	413.40 ± 3.65
Vanillic acid	-	45.00 ± 0.88	-
Syringic acid	4.38 ± 0.12	72.22 ± 0.62	1.69 ± 0.01
Epicatechin	513.52 ± 2.48	425.78 ± 4.22	278.90 ± 2.33
p-Coumaric acid	-	<0.02	-
Hyperoside	<0.02	-	<0.02
Isoquercitrin	29.70 ± 0.30	6.59 ± 0.09	3.50 ± 0.05
Rutin	2.63 ± 0.07	-	2.63 ± 0.04
Quercitrin	26.08 ± 0.37	-	-
Quercetin	6.69 ± 0.02	9.99 ± 0.01	-
Luteolin	3.50 ± 0.04	-	-

Each value is the mean ± SD of three independent measurements. WP—white pomace, RP—red pomace, C—canes. Values are the mean ± SD (*n* = 3). Note: “-” means not found, below the detection limit.

**Table 3:** The antimicrobial potential of WPE, RPE, CE, and CoE: the diameter of inhibition zone (mm).

	Inhibition Diameter for the Tested Extracts (mm ± SD)				
Bacterial Strain	WPE	RPE	CE	CoE	Control
*Streptococcus mutans* ATCC 25175	12 ± 0.71	11 ± 0.71	15 ± 1.41	13 ± 0.71	0 ± 0.00
*Porphyromonas gingivalis* ATCC 33277	11 ± 0.71	11 ± 0.71	10 ± 0.71	13 ± 0.00	0 ± 0.00
*Enterococcus faecalis* ATCC 29212	11 ± 0.71	10 ± 0.00	12 ± 0.00	10 ± 0.00	0 ± 0.00
*Escherichia coli* ATCC 25922	10 ± 0.00	11 ± 0.00	10 ± 0.00	13 ± 1.41	0 ± 0.00
*Staphylococcus aureus* ATCC 25923	10 ± 0.00	11 ± 0.00	10 ± 0.71	11 ± 0.71	0 ± 0.00
*Klebsiella* spp.	10 ± 0.00	11 ± 0.71	10 ± 0.71	11 ± 0.71	0 ± 0.00
*Citrobacter* spp.	10 ± 0.00	9 ± 0.00	0 ± 0.00	9 ± 0.00	0 ± 0.00

Legend: Control = aqueous ethanol solution of 50% (*V*/*V*).

**Table 4:** Texture parameters of the freeze-dried mouthwashes.

	Hardness (g)	Rigidity (g)	Fracturability (g)
F1	91.00 ± 25.70	80.70 ± 21.0	41.30 ± 42.60
F2	249.50 ± 36.30 *	192.50 ± 29.70 *	138.80 ± 55.40
F3	35.80 ± 3.40 *	28.80 ± 2.90 *	13.30 ± 1.00
F4	135.30 ± 38.30	96.80 ± 6.50	54.20 ± 29.80
F5	451.70 ± 198.80 *	304.20 ± 92.60 *	133.80 ± 36.20 *

Results are presented as mean ± standard deviation of three measurements. “*” indicates statistically significant differences (*p* < 0.05, ANOVA single factor test) between the formulations F2–F4 and F1.

**Table 5:** Texture parameters of the reconstituted mouthwashes.

	Reconstitution Time (s)	Texture Analysis			Viscosity (cPs)
		Hardness (g)	Adhesive Force (g)	Stringiness Length (mm)	
F1	30.56 ± 1.65	14.20 ± 0.60	9.50 ± 0.50	0.54 ± 0.06	5.65 ± 0.04
F2	9.04 ± 1.60 *	15.20 ± 0.80	9.30 ± 0.30	0.54 ± 0.06	7.92 ± 0.02 *
F3	4.82 ± 0.76 *	13.70 ± 1.30	10.00 ± 1.00	0.53 ± 0.06	5.48 ± 0.04
F4	6.95 ± 0.96 *	14.20 ± 0.30	9.30 ± 0.30	0.53 ± 0.06	5.55 ± 0.02
F5	8.81 ± 1.17 *	17.00 ± 1.80	7.70 ± 0.30	0.53 ± 0.06	8.09 ± 0.05 *
F6	-	15.30 ± 0.30	7.30 ± 0.30	0.51 ± 0.01	7.85 ± 0.01 *

Results are presented as mean ± standard deviation of three measurements. “*” indicates statistically significant differences (*p* < 0.05, ANOVA single factor test) between the formulations F2–F6 and F1. F6, Commercial mouthwash Sensodyne Cool Mint.

## Data Availability

Data is contained within the article.

## References

[1] Rasines-Perea Z., Teissedre P.-L. (2017). Grape Polyphenols’ Effects in Human Cardiovascular Diseases and Diabetes. Molecules.

[2] Georgiev V., Ananga A., Tsolova V. (2014). Recent advances and uses of grape flavonoids as nutraceuticals. Nutrients.

[3] Xia E.-Q., Deng G.-F., Guo Y.-J., Li H.-B. (2010). Biological activities of polyphenols from grapes. Int. J. Mol. Sci..

[4] Ky I., Teissedre P.L. (2015). Characterisation of Mediterranean grape pomace seed and skin extracts: Polyphenolic content and antioxidant activity. Molecules.

[5] Kalekhan F., Bala N., Rao S., Pais M.L.J., Adnan M., Sajan S., Baliga M.S., Kabir Y. (2020). Usefulness of Grape Seed Polyphenols in the Prevention of Skin Cancer: A Mini Review.

[6] Furiga A., Lonvaud-Funel A., Badet C. (2009). In vitro study of antioxidant capacity and antibacterial activity on oral anaerobes of a grape seed extract. Food Chem..

[7] Crisan D., Crisan M., Moldovan M., Lupsor M., Badea R. (2012). Ultrasonographic assessment of the cutaneous changes induced by topical flavonoid therapy. Clin. Cosmet. Investig. Dermatol..

[8] Nichols J.A., Katiyar S.K. (2010). Skin photoprotection by natural polyphenols: Anti-inflammatory, antioxidant and DNA repair mechanisms. Arch. Dermatol. Res..

[9] Soto M., Falqué E., Domínguez H. (2015). Relevance of natural phenolics from grape and derivative products in the formulation of cosmetics. Cosmetics.

[10] Moldovan M.L., Carpa R., Fizeșan I., Vlase L., Bogdan C., Iurian S.M., Benedec D., Pop A. (2020). Phytochemical Profile and Biological Activities of Tendrils and Leaves Extracts from a Variety of *Vitis vinifera* L.. Antioxidants.

[11] Saleem H.G.M., Seers C.A., Sabri A.N., Reynolds E.C. (2016). Dental plaque bacteria with reduced susceptibility to chlorhexidine are multidrug resistant. BMC Microbiol..

[12] Palombo E.A. (2011). Traditional Medicinal Plant Extracts and Natural Products with Activity against Oral Bacteria: Potential Application in the Prevention and Treatment of Oral Diseases. Evid. Based Complement. Alternat. Med..

[13] Ferri M., Vannini M., Ehrnell M., Eliasson L., Xanthakis E., Monari S., Sisti L., Marchese P., Celli A., Tassoni A. (2020). From winery waste to bioactive compounds and new polymeric biocomposites: A contribution to the circular economy concept. J. Adv. Res..

[14] Wu C.D. (2009). Grape Products and Oral Health. J. Nutr..

[15] Costalonga M., Herzberg M.C. (2014). The oral microbiome and the immunobiology of periodontal disease and caries. Immunol. Lett..

[16] Luchian C.E., Cotea V.V., Vlase L., Toiu A.M., Colibaba L.C., Raschip I.E., Nadas G., Gheldiu A.M., Tuchilus C., Rotaru L. (2019). Antioxidant and antimicrobial effects of grape pomace extracts. BIO Web Conf..

[17] Moldovan M.L., Iurian S., Puscas C., Silaghi-Dumitrescu R., Hanganu D., Bogdan C., Vlase L., Oniga I., Benedec D. (2019). A design of experiments strategy to enhance the recovery of polyphenolic compounds from *Vitis vinifera* by-products through heat reflux extraction. Biomolecules.

[18] Pop A., Drugan T., Gutleb A.C., Lupu D., Cherfan J., Loghin F., Kiss B. (2016). Individual and combined in vitro (anti)androgenic effects of certain food additives and cosmetic preservatives. Toxicol. In Vitro.

[19] Rusu M.E., Fizesan I., Pop A., Mocan A., Gheldiu A.-M., Babota M., Vodnar D.C., Jurj A., Berindan-Neagoe I., Vlase L. (2020). Walnut (*Juglans regia* L.) Septum: Assessment of Bioactive Molecules and In Vitro Biological Effects. Molecules.

[20] Atlas R.M. (2010). Handbook of Microbiological Media.

[21] Carpa R., Dragan-Bularda M., Muntean V. (2014). Microbiologie Generala—Lucrari Practice.

[22] Trinh T. (2012). On the texture profile analysis test. Qual. Life Chem. Eng..

[23] Brookfield Engineering Laboratories Ltd. CT3 Texture Analyzer Operating Instructions.

[24] Nirmala J.G., Celsia S.E., Swaminathan A., Narendhirakannan R.T., Chatterjee S. (2018). Cytotoxicity and apoptotic cell death induced by *Vitis vinifera* peel and seed extracts in A431 skin cancer cells. Cytotechnology.

[25] Giovannelli L., Innocenti M., Santamaria A.R., Bigagli E., Pasqua G., Mulinacci N. (2014). Antitumoural activity of viniferin-enriched extracts from *Vitis vinifera* L. cell cultures. Nat. Prod. Res..

[26] De Sales N.F.F., Silva da Costa L., Carneiro T.I.A., Minuzzo D.A., Oliveira F.L., Cabral L., Torres A.G., El-Bacha T. (2018). Anthocyanin-rich grape pomace extract (*Vitis vinifera* L.) from wine industry affects mitochondrial bioenergetics and glucose metabolism in human hepatocarcinoma HepG2 cells. Molecules.

[27] Loizzo M.R., Sicari V., Pellicanò T., Xiao J., Poiana M., Tundis R. (2019). Comparative analysis of chemical composition, antioxidant and anti-proliferative activities of Italian *Vitis vinifera* by-products for a sustainable agro-industry. Food Chem. Toxicol..

[28] Moldovan M.L., Bogdan C., Iurian S., Roman C., Oniga I., Benedec D. (2020). Phenolic content and antioxidant capacity of pomace and canes extracts of some *Vitis vinifera* varieties cultivated in Romania. Farmacia.

[29] Milinčić D.D., Stanisavljević N.S., Kostić A.Ž., Bajić S.S., Kojić M.O., Gašić U.M., Barać M.B., Stanojević S.P., Tešić Ž.L., Pešić M.B. (2021). Phenolic compounds and biopotential of grape pomace extracts from Prokupac red grape variety. LWT.

[30] Maluf F.D., Gonçalves M.M., D’Angelo R.W.O., Girassol A.B., Tulio A.P., Pupo Y.M., Farago P.V. (2018). Cytoprotection of antioxidant biocompounds from grape pomace: Further exfoliant phytoactive ingredients for cosmetic products. Cosmetics.

[31] Fernández-Fernández A.M., Iriondo-DeHond A., Nardin T., Larcher R., Dellacassa E., Medrano-Fernandez A., Castillo M.D. (2020). In Vitro Bioaccessibility of Extractable Compounds from Tannat Grape Skin Possessing Health Promoting Properties with Potential to Reduce the Risk of Diabetes. Foods.

[32] Pervin M., Hasnat M., Lee Y.M., Kim D.H., Jo J.E., Lim B.O. (2014). Antioxidant activity and acetylcholinesterase inhibition of grape skin anthocyanin (GSA). Molecules.

[33] Anastasiadi M., Pratsinis H., Kletsas D., Skaltsounis A.-L., Haroutounian S.A. (2012). Grape stem extracts: Polyphenolic content and assessment of their in vitro antioxidant properties. LWT Food Sci. Technol..

[34] Goutzourelas N., Stagos D., Spanidis Y., Liosi M., Apostolou A., Priftis A., Haroutounian S., Spandidos D.A., Tsatsakis A.M., Kouretas D. (2015). Polyphenolic composition of grape stem extracts affects antioxidant activity in endothelial and muscle cells. Mol. Med. Rep..

[35] Marabini L., Melzi G., Lolli F., Dell’Agli M., Piazza S., Sangiovanni E., Marinovich M. (2020). Effects of *Vitis vinifera* L. leaves extract on UV radiation damage in human keratinocytes (HaCaT). J. Photochem. Photobiol. B Biol..

[36] Nirmala J.G., Narendhirakannan R.T. (2011). In vitro antioxidant and antimicrobial activities of grapes (*Vitis vinifera* L.) seed and skin extracts–Muscat variety. Int. J. Pharm. Pharm. Sci..

[37] Haber J., Wattles J., Crowley M., Mandell R., Joshipura K., Kent R.L. (1993). Evidence for cigarette smoking as a major risk factor for periodontitis. J. Periodontol..

[38] Katono T., Kawato T., Tanabe N., Tanaka H., Suzuki N., Kitami S., Morita T., Motohashi M., Maeno M. (2009). Effects of nicotine and lipopolysaccharide on the expression of matrix metalloproteinases, plasminogen activators, and their inhibitors in human osteoblasts. Arch. Oral Biol..

[39] Tanaka H., Tanabe N., Shoji M., Suzuki N., Katono T., Sato S., Motohashi M., Maeno M. (2006). Nicotine and lipopolysaccharide stimulate the formation of osteoclast-like cells by increasing macrophage colony-stimulating factor and prostaglandin E2 production by osteoblasts. Life Sci..

[40] Almasri A., Wisithphrom K., Windsor L.J., Olson B. (2007). Nicotine and lipopolysaccharide affect cytokine expression from gingival fibroblasts. J. Periodontol..

[41] Yarahmadi A., Zal F., Bolouki A. (2017). Protective effects of quercetin on nicotine induced oxidative stress in ‘HepG2 cells. Toxicol. Mech. Methods.

[42] Balakrishnan A., Menon V.P. (2007). Antioxidant properties of hesperidin in nicotine-induced lung toxicity. Fundam. Clin. Pharmacol..

[43] Paunović M.G., Ognjanović B.I., Matić M.M., Štajn A.Š., Saičić Z.S. (2016). Protective effects of quercetin and vitamin C against nicotine-induced toxicity in the blood of Wistar rats. Arch. Ind. Hyg. Toxicol..

[44] Warnakulasuriya S.N., Rupasinghe H.P.V. (2016). Novel long chain fatty acid derivatives of quercetin-3-*O*-glucoside reduce cytotoxicity induced by cigarette smoke toxicants in human fetal lung fibroblasts. Eur. J. Pharmacol..

[45] Li L., Sun W., Wu T., Lu R., Shi B. (2017). Caffeic acid phenethyl ester attenuates lipopolysaccharide-stimulated proinflammatory responses in human gingival fibroblasts via NF-κB and PI3K/Akt signaling pathway. Eur. J. Pharmacol..

[46] Desjardins J., Grenier D. (2012). Neutralizing effect of green tea epigallocatechin-3-gallate on nicotine-induced toxicity and chemokine (C-C motif) ligand 5 secretion in human oral epithelial cells and fibroblasts. J. Investig. Clin. Dent..

[47] Fraternale D., Rudov A., Prattichizzo F., Olivieri F., Ricci D., Giacomini E., Carloni S., Azzolini C., Gordillo B., Jara-Palacios M.J. (2016). Chemical composition and “in vitro” anti-inflammatory activity of *Vitis vinifera* L. (var. Sangiovese) tendrils extract. J. Funct. Foods.

[48] Millan-Linares M.C., Bermudez B., Martin M.E., Muñoz E., Abia R., Millan F., Muriana F.J.G., Montserrat-de la Paz S. (2018). Unsaponifiable fraction isolated from grape (*Vitis vinifera* L.) seed oil attenuates oxidative and inflammatory responses in human primary monocytes. Food Funct..

[49] Singh J., Singh A.K., Singh A. (2009). Analgesic and anti-inflammatory activity of methanolic extract of *Vitis vinifera* leaves. Pharmacologyonline.

[50] Filip A., Clichici S., Daicoviciu D., Catoi C., Bolfa P., Postescu I.D., Gal A., Baldea I., Gherman C., Muresan A. (2011). Chemopreventive effects of Calluna vulgaris and *Vitis vinifera* extracts on UVB-induced skin damage in SKH-1 hairless mice. J. Physiol. Pharmacol..

[51] Mueller H., Eick S., Moritz A., Lussi A., Gruber R. (2017). Cytotoxicity and Antimicrobial Activity of Oral Rinses In Vitro. Biomed. Res. Int..

[52] Kouidhi B., Al Qurashi Y.M.A., Chaieb K. (2015). Drug resistance of bacterial dental biofilm and the potential use of natural compounds as alternative for prevention and treatment. Microb. Pathog..

[53] Hashim N., Paramasivam M., Tan J.S., Kernain D., Hussin M.H., Brosse N., Gambier F., Raja P.B. (2020). Green mode synthesis of silver nanoparticles using Vitis vinifera’s tannin and screening its antimicrobial activity/apoptotic potential versus cancer cells. Mater. Today Commun..

[54] Yadav D., Kumar A., Kumar P., Mishra D. (2015). Antimicrobial properties of black grape (*Vitis vinifera* L.) peel extracts against antibiotic-resistant pathogenic bacteria and toxin producing molds. Indian J. Pharmacol..

[55] Friedman M. (2014). Antibacterial, antiviral, and antifungal properties of wines and winery byproducts in relation to their flavonoid content. J. Agric. Food Chem..

[56] Iurian S., Bogdan C., Tomuță I., Szabó-Révész P., Chvatal A., Leucuța S.E., Moldovan M., Ambrus R. (2017). Development of oral lyophilisates containing meloxicam nanocrystals using QbD approach. Eur. J. Pharm. Sci..

[57] Rowe R.C., Sheskey P., Quinn M. (2009). Handbook of Pharmaceutical Excipients.

[58] Harrison Cole B., Kroeger D.C., Wilson M.E. (1990). Non-Alcoholic Mouthwash. U.S. Patent.

[59] Singh M., Sharma D., Kumar D., Singh G., Swami G., Rathore M.S. (2020). Formulation, Development, and Evaluation of Herbal Effervescent Mouthwash Tablet Containing Azadirachta Indica (Neem) and Curcumin for the Maintenance of Oral Hygiene. Recent Pat. Drug Deliv. Formul..

[60] Hurwitz M.M. (2014). Oral Hygiene Tablets and Capsules for Direct Oral Delivery of Active Ingredients. U.S. Patents.

[61] Mumoli J.A. (2003). Single-Dose Quick-Dissolving Cleansing Agent with Medicinal Properties. U.S. Patents.

[62] Bhatta S., Stevanovic Janezic T., Ratti C. (2020). Freeze-Drying of Plant-Based Foods. Foods.

[63] Chikwanha O.C., Raffrenato E., Opara U.L., Fawole O.A., Setati M.E., Muchenje V., Mapiye C. (2018). Impact of dehydration on retention of bioactive profile and biological activities of different grape (*Vitis vinifera* L.) pomace varieties. Anim. Feed Sci. Technol..

[64] Costa J.S.R., de Oliveira Cruvinel K., Oliveira-Nascimento L. (2019). A mini-review on drug delivery through wafer technology: Formulation and manufacturing of buccal and oral lyophilizates. J. Adv. Res..

